# Nomograms for prognostic factors of spinal giant cell tumor combining traditional clinical characteristics with inflammatory biomarkers after gross total resection

**DOI:** 10.18632/oncotarget.21168

**Published:** 2017-09-22

**Authors:** Jialin Li, Bo Li, Pingting Zhou, Jian Zhao, Zhipeng Wu, Xinghai Yang, Haifeng Wei, Tianrui Chen, Jianru Xiao

**Affiliations:** ^1^ Department of Bone Tumor Surgery, Changzheng Hospital, Second Military Medical University, Shanghai, China; ^2^ Department of Oncology, Shanghai Ninth People's Hospital, Shanghai Jiaotong University School of Medicine, Shanghai, China

**Keywords:** giant cell tumor, spine, inflammatory biomarkers, disease-free survival, nomogram

## Abstract

Giant cell tumor (GCT) of bone is a common primary bone tumor, which exhibits local aggressiveness and recurrent potential, especially for the spinal lesion. Increasing evidence indicates that inflammation plays a vital role in tumorigenesis and progression. The prognostic value of inflammatory biomarkers in GCT has not been established. A retrospective analysis was conducted in patients with spinal GCT in Changzheng Hospital Orthopedic Oncological Center (CHOOC) between January 2005 and October 2015 and 129 patients were identified eligible. Traditional clinical parameters and inflammatory indexes such as Neutrophil-to-lymphocyte ratio (NLR), platelet-to-lymphocyte ratio (PLR), lymphocyte-to-monocyte ratio (LMR), and albumin/globulin ratio (AGR) were concluded and analyzed. Kaplan-Meier analysis was used to calculate the disease-free survival (DFS). Cox regression analysis was performed to assess the prognostic factors. Nomograms were established to predict DFS quantitatively for the first time, and Harrell’s concordance index (c-index) was adopted to evaluate prediction accuracy. As results, the DFS was 78.3% in the cohort. Patients were stratified into 2 groups by NLR (≤ 2.70 and > 2.70), PLR (≤ 215.80 and > 215.80), LMR (≤ 2.80 and >2.80) and AGR (< 1.50 and ≥ 1.50). Patients with NLR > 2.70, PLR > 215.80, LMR ≤ 2.80 and AGR < 1.50 were significantly associated with decreased DFS (*p* < 0.05). Multivariate analysis indicated that treatment history, tumor length, bisphosphonate treatment, NLR and PLR were independent factors of DFS (*p* < 0.05, respectively). In addition, nomogram on DFS was established according to all significant factors, and c-index was 0.728 (95% CI: 0.710-0.743). Nomograms based on DFS can be recommended as practical models to evaluate prognosis for spinal GCT patients.

## INTRODUCTION

Giant cell tumor (GCT) of bone is a common primary bone tumor and usually occurs in patients aged 20–40 years [1, 2]. Spine is a relatively rare site for GCT, accounting for 1.4–9.4% [3]. GCT is predominantly regarded as a benign lesion, but it exhibits local aggressiveness and recurrent potential [4–6]. Surgical resection is the fundamental treatment option for GCT in the spine and gross total resection (GTR) realized by en bloc or piece-meal method is the first choice [3, 7]. However, spinal GCT poses difficulty for the surgeon owing to its proximity to vital neurovascular structures. The postoperative recurrence rate of spinal GCT, which ranges from 20% to 50%, is much higher than the lesion in the extremities [3, 7, 8], even though the GTR is conducted. Recurrence might exacerbate the neurologic defects, cause malignant transformation or distant metastasis, and even lead to death. Thus, a better model to predict the prognosis of spinal GCT patients after GTR is urgently needed.

Our center has published several parameters for predicting local recurrence including traditional clinical factors such as surgical method and bisphosphonate treatment [3, 5, 9]. However, the recurrent rate or disease-free survival vary widely even in patients with same therapeutic process [7, 10, 11]. Therefore, easier and more accurate parameters able to predict the patient prognosis is required.

Recent reports revealed that tumor progression and prognosis is determined not only by tumor characteristics but also by the host inflammatory response [12–16]. It has increasingly been recognized that tumor infiltrating inflammatory cells are responsible for producing inflammatory mediators and cytokines that induce angiogenesis, tumor growth, invasion and metastasis [17–19]. Accordingly, serum white blood cells, neutrophils, lymphocytes, platelets and acute-phase proteins, such as C-reactive protein and albumin, have been evaluated in different tumors and found to predict for prognosis and response to treatment [20]. These parameters are simple and easy to measure using widely applied and standardized assays. Moreover, a series of combinations of these factors, such as neutrophil-to-lymphocyte ratio (NLR), platelet-to-lymphocyte ratio (PLR), lymphocyte-to-monocyte ratio (LMR) and albumin/globulin ratio (AGR), have been performed to evaluate the prognosis in various cancers, such as breast cancer, lung adenocarcinoma, colorectal cancer and gastric cancer [21–24]. However, no study has taken the inflammatory parameter into consideration to predict the prognosis of the GCT. Because GCT is mostly regarded as a benign lesion, the mortality caused by GCT is not common; what’s more, the clinical adverse events are usually caused by local recurrence. Thus, our focus is concentrated on the recurrent rate that is the disease free survival (DFS) rather than the overall survival (OS).

There are many studies in which the development of nomograms leads to a successful application for oncology prognosis [25–28]. Nomograms for predicting follow-up outcome for GCT are scarce. Our primary goal was to use nomograms to comprehensively investigate the prognostic role of traditional clinical characteristics as well as inflammatory biomarkers (NLR, PLR, LMR and AGR) after gross total resection in patients with spinal GCT.

## RESULTS

### Patients’ baseline characteristics

The characteristics of 129 patients were shown in Table 1. The series was comprised of 55 men and 74 women, with a mean age of 33.5 (range 11–69) years. Of these patients, 101 were admitted for primary GCT, and the remaining 28 were recurrent after surgical treatment performed 1 in other institutions. Lesions were detected in the cervical spine (36), thoracic spine (44), lumbar spine (22), and sacrum (27) (Table 1). The mean follow-up period was 68.6 months (range 18–155). Recurrence was detected in 28 patients after initial surgery in our center, while death occurred in 7 cases. The mean time from surgery to recurrence was 15.2 months (range 2–41), and 21 patients (78.6 %) developed recurrence within 24 months.

**Table 1:** Clinical data for 129 cases of GCT in the spine

	Clinical factors	Counts (%)
Age		33.5 ± 12.8
Gender	Male	55 (42.6%)
	Female	74 (57.4%)
Treatment history	Primary	101 (78.3%)
	Recurrent	28 (21.7%)
Location	Cervical spine	36 (27.9%)
	Thoracic spine	44 (34.1%)
	Lumbar spine	22 (17.1%)
	Sacral spine	27 (20.9%)
No. of involved segment	Monosegment	80 (62%)
	Multisegmen	49 (38%)
Preoperative Frankel score	A-C	41(31.8%)
	D-E	88 (68.2%)
Resection mode	Piecemeal	97 (75.2%)
	*En bloc*	32 (24.8%)
Bisphosphonate treatment	Yes	77 (59.7%)
	No	52 (40.3%)
Adjuvant radiotherapy	Yes	53 (41.1%)
	No	76 (58.9%)
Recurrence	Yes	28 (21.7%)
	No	101 (78.3%)
Death	Yes	7 (5.4%)
	No	122 (94.6%)

GCT: giant cell tumor

### Identification of NLR, PLR, LMR and AGR optimal cut-off values

**Figure 1:**
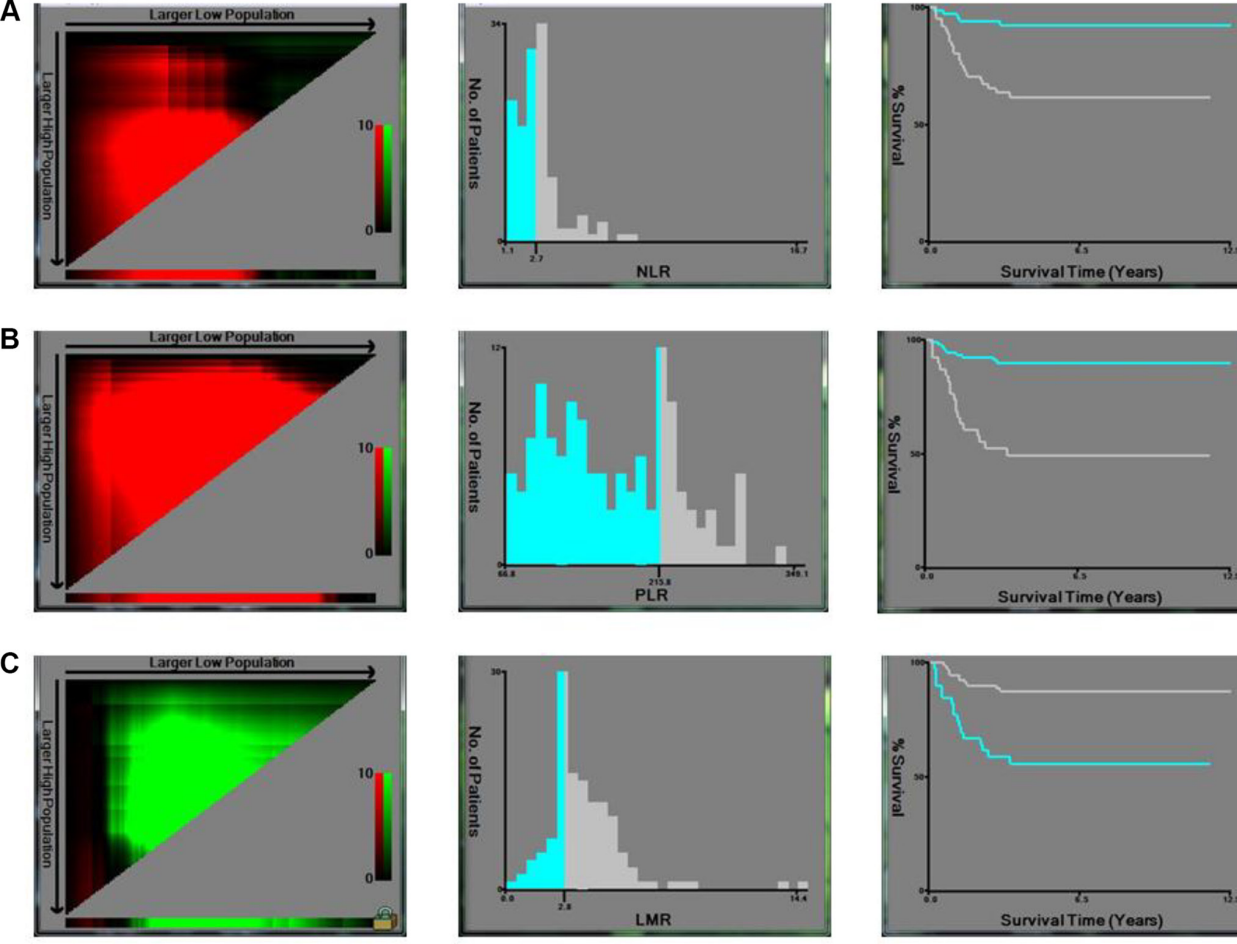
X-tile analyses of DFS were performed using patients’ data to determine the optimal cut-off values for NLR, PLR and LMR The sample of GCT patients was equally divided into training and validation sets. X-tile plots of training sets are shown in the left panels, with plots of matched validation sets shown in the smaller inset. The optimal cut-off values highlighted by the black circles in left panels are shown in histograms of the entire cohort (middle panels), and Kaplan–Meier plots are displayed in right panels. *p* values were determined by using the cut-off values defined in training sets and applying them to validation sets. The optimal cut-off values for NLR, PLR and LMR of DFS were 2.70, 215.80, and 2.80 respectively.

X-tile program was used to determine the optimal cut-off values for NLR, PLR and LMR of DFS, which were 2.7, 215.8 and 2.8, respectively (Figure 1). The chi-square log-rank value of NLR, PLR and LMR were 7.79, 36.59 and 63.33, respectively. Patients were divided into two groups for further analysis (NLR ≤ 2.70 and >2.70; PLR ≤ 215.80 and > 215.80; LMR ≤ 2.80 and > 2.80). The cut-off value for AGR was 1.50, according to the standard value reported by Clinical Laboratory Department in Changzheng Hospital and patients were then divided into two groups (AGR < 1.50 and ≥ 1.50). Kaplan–Meier survival analysis revealed that NLR > 2.70, PLR > 215.80, LMR ≤ 2.80 and AGR < 1.50 were significantly associated with decreased DFS (*p* < 0.05).

The patients’ baseline characteristics and patients’ clinical parameters stratified by NLR, PLR, LMR and AGR are described in Table 2. Multivariate logistic regression analysis showed that NLR was associated with age, gender and tumor length (*p* < 0.05); PLR was associated with age and bisphosphonate treatment (*p* < 0.05); LMR was associated with treatment history and bisphosphonate treatment (*p* < 0.05) even after being adjusted for other statistically significant factors in chi-square tests (*p* < 0.05, Table 3).

**Table 2:** The patients’ baseline characteristics and patients’ clinical parameters stratified by NLR, PLR, LMR and AGR

	Case	NLR n (%)			PLR n (%)			LMR n (%)			AGR n (%)		
	NO.(100%)	≤ 2.7	> 2.7	*p*	≤ 215.8	> 215.8	*p*	≤ 2.8	> 2.8	*p*	< 1.5	≥ 1.5	*p*
**Age (years)**				0.002*			0.003*			0.023*			0.216
≤ 60	116 (89.9)	61 (52.6)	55 (47.4)		86 (74.1)	30 (25.9)		34 (29.3)	82 (70.7)		62 (53.4)	54 (46.6)	
> 60	13 (10.1)	1 (7.7)	12 (92.3)		4 (30.8)	9 (69.2)		8 (61.5)	5 (38.5)		9 (69.2)	4 (30.8)	
**Gender**				0.035*			0.112			0.097			0.263
Male	74 (57.4)	30 (40.5)	44 (59.5)		48 (64.9)	26 (35.1)		28 (37.8)	46 (62.2)		43 (58.1)	31 (41.9)	
Female	55 (42.6)	32 (58.2)	23 (41.8)		42 (76.4)	13 (23.6)		14 (25.5)	41 (74.5)		28 (50.9)	27 (49.1)	
**Treatment history**				0.202			0.486			0.025*			0.489
Primary	101 (78.3)	51 (50.5)	50 (49.5)		71 (70.3)	30 (29.7)		28 (27.7)	73 (72.3)		55 (54.5)	46 (45.5)	
Recurrent	28 (21.7)	11 (39.3)	17 (60.7)		19 (67.9)	9 (32.1)		14 (50.0)	14 (50.0)		16 (57.1)	12 (42.9)	
**Duration of symptoms**				0.101			0.028*			0.100			0.046*
≤ 12	32 (24.8)	19 (59.4)	13 (40.6)		27 (84.4)	34 (15.6)		7 (21.9)	25 (78.1)		13 (40.6)	19 (59.4)	
> 12	97 (75.2)	43 (44.3)	54 (55.7)		63 (64.9)	9 (35.1)		35 (36.1)	62 (63.9)		58 (59.8)	39 (40.2)	
**Preoperative Frankel score**				0.325			0.479			0.102			0.511
A-C	41 (31.8)	18 (43.9)	23 (56.1)		28 (68.3)	13 (31.7)		17 (41.5)	24 (58.5)		23 (56.1)	18 (43.9)	
D-E	88 (68.2)	44 (50.0)	44 (50.0)		62 (70.5)	26 (29.5)		25 (28.4)	63 (71.6)		48 (54.5)	40 (45.5)	
**Tumor location**				0.631			0.657			0.246			0.626
Cervical	36 (27.9)	16 (44.4)	20 (55.6)		25 (69.4)	11 (30.6)		12 (33.3)	24 (66.7)		20 (55.6)	16 (45.7)	
Thoracic	44 (34.1)	20 (45.5)	24 (54.5)		32 (72.7)	12 (27.3)		15 (34.1)	29 (65.9)		25 (56.8)	19 (43.2)	
Lumbar	22 (17.1)	10 (45.5)	12 (54.5)		13 (59.1)	9 (40.9)		10 (45.5)	12 (54.5)		13 (59.1)	9 (40.9)	
Sacrum	27 (20.9)	16 (59.3)	11 (40.7)		20 (74.1)	7 (25.9)		5 (18.5)	22 (81.5)		13 (48.1)	14 (51.9)	
**Involved segment**				0.365			0.453			0.432			0.318
Monosegment	80 (62.0)	37 (46.2)	43 (53.8)		55 (68.8)	25 (31.3)		27 (33.8)	53 (66.3)		46 (57.5)	34 (42.5)	
Multisegment	49 (38.0)	25 (51.0)	24 (49.0)		35 (71.4)	14 (28.6)		15 (30.6)	34 (69.4)		25 (51.0)	24 (49.0)	
Tumor length (cm)				0.015*			0.009*			0.054			0.087
≤ 3	96 (74.4)	52 (54.2)	44 (45.8)		73 (76.0)	23 (24.0)		27 (28.1)	69 (71.9)		49 (51.01)	47 (49.0)	
> 3	33 (25.6)	10 (30.3)	23 (69.7)		17 (51.5)	16 (48.5)		15 (45.5)	18 (54.5)		22 (66.7)	11 (33.3)	
**Preoperative embolization**				0.469			0.417			0.352			0.385
Yes	66 (51.2)	31 (47.0)	35 (53.0)		45 (68.2)	21 (31.8)		23 (34.8)	43 (65.2)		35 (53.0)	31 (47.0)	
No	63 (48.8)	31 (49.2)	32 (50.8)		45 (71.4)	18 (28.6)		19 (30.2)	44 (69.8)		36 (57.1)	27 (42.9)	
**Enneking staging**				0.259			0.604			0.631			0.478
I	15 (11.6)	5 (33.3)	10 (66.7)		11 (73.3)	4 (26.7)		6 (40.0)	9 (60.0)		7 (46.7)	8 (53.3)	
II	78 (60.5)	38 (48.7)	40 (51.3)		55 (70.5)	23 (29.5)		23 (29.5)	55 (70.5)		43 (55.1)	35 (44.9)	
III	36 (27.9)	19 (52.8)	17 (47.2)		24 (66.7)	12 (33.3)		13 (36.1)	23 (63.9)		21 (58.3)	15 (41.7)	
**Bisphosphonate treatment**				0.018*			0.008*			0.001*			0.033*
Yes	72 (55.8)	41 (56.9)	31 (43.1)		57 (79.2)	15 (20.8)		15 (20.8)	57 (79.2)		34 (47.2)	38 (52.8)	
No	57 (44.2)	21 (36.8)	36 (63.2)		33 (57.9)	24 (42.1)		27 (47.4)	30 (52.6)		37 (64.9)	20 (35.1)	
**Adjuvant radiotherapy**				0.356			0.421			0.461			0.453
Yes	53 (41.1)	27 (50.9)	26 (49.1)		38 (71.7)	15 (28.3)		18 (34.0)	35 (66.0)		30 (56.6)	23 (43.4)	
No	76 (58.9)	35 (46.1)	41 (53.9)		52 (68.4)	24 (31.6)		24 (31.6)	52 (68.4)		41 (53.9)	35 (46.1)	

NLR: neutrophil-to-lymphocyte ratio; PLR: platelet-to-lymphocyte ratio; LMR: lymphocyte-to-monocyte ratio; AGR: albumin/globulin ratio

*: *p* value ≤ 0.05.

**Table 3:** Multivariate logistic regression analysis of inflammatory index

	NLR		PLR		LMR		AGR	
	OR (95%)	*p*	OR (95%)	*p*	OR (95%)	*p*	OR (95%)	*p*
**Age (years)**		0.011*		0.004*		0.106		0.419
≤ 60	1.000		1.000		1.000		1.000	
> 60	11.246 (1.170–108.126)		10.467 (1.943–56.379)		4.546 (0.059–1.342)		0.548 (0.124–2.411)	
**Gender**		0.024*		0.330		0.272		0.679
Male	1.000		1.000		1.000		1.000	
Female	0.373 (0.156–0.896)		.617 (0.231–1.644)		1.717 (0.648–4.546)		1.189 (0.524–2.700)	
**Treatment history**		0.379		0.585		0.023*		0.143
Primary	1.000		1.000		1.000		1.000	
Recurrent	1.599 (0.558–4.581)		1.371 (0.443–4.243)		0.288 (0.096–0.862)		0.725 (0.265–1.982)	
**Duration of symptoms**		0.218		0.052		0.579		0.074
≤ 12	1.000		1.000		1.000		1.000	
> 12	2.022 (0.651–6.282)		3.689 (0.928–14.657)		0.700 (0.197–2.485)		0.384 (0.132–1.116)	
**Preoperative Frankel score**		0.873		0.596		0.315		0.795
A–C	1.000		1.000		1.000		1.000	
D–E	0.929 (0.375–2.298)		0.760 (0.275–2.100)		1.637 (0.625–4.290)		0.891 (0.375–2.120)	
**Tumor location**		0.888		0.380		0.611		0.415
Cervical	1.000		1.000		1.000		1.000	
Thoracic	0.625 (0.102–3.832)		5.058 (0.568–45.084)		0.989 (0.120–8.162)		0.514 (0.093–2.843)	
Lumbar	0.631 (0.156–2.556)		1.313 (0.255–6.771)		1.797 (0.358–9.031)		1.507 (0.404–5.626)	
Sacrum	0.942 (0.194–4.580)		1.466 (0.242–8.881)		2.400 (0.406–14.185)		0.734 (0.159–3.385)	
**Involved segment**		0.770		0.856		0.130		0.810
Monosegment	1.000		1.000		1.000		1.000	
Multisegment	1.166 (0.417–3.256)		1.115 (0.346–3.595)		0.416 (0.132–1.314)		1.126 (0.429–2.953)	
**Tumor length (cm)**		0.042*		0.073		0.146		0.143
≤ 3	1.000		1.000		1.000		1.000	
> 3	2.798 (1.039–7.539)		2.447 (0.922–6.493)		0.475 (0.174–1.295)		0.506 (0.200–1.278)	
**Preoperative embolization**		0.772		0.771		0.505		0.424
Yes	1.000		1.000		1.000		1.000	
No	1.159 (0.428–3.141)		1.176 (0.395–3.496)		1.452 (0.482–4.374)		0.676 (0.258–1.770)	
**Enneking staging**		0.420		0.127		0.451		0.182
I	1.000		1.000		1.000		1.000	
II	0.534 (0.103–2.757)		8.037 (0.901–71.705)		0.312 (0.046–2.137)		0.273 (0.055–1.351)	
III	0.425 (0.116–1.562)		1.776 (0.454–6.947)		0.514 (0.131–2.015)		0.946 (0.285–3.144)	
**Bisphosphonate treatment**		0.153		0.029*		0.003*		0.068
Yes	1.000		1.000		1.000		1.000	
No	1.905 (0.783–4.634)		2.949 (1.098–7.917)		0.234 (0.086–0.640)		0.449 (0.188–1.072)	
**Adjuvant radiotherapy**		0.827		0.386		0.573		0.843
Yes	1.000		1.000		1.000		1.000	
No	1.104 (0.455–2.678)		1.565 (0.566–4.322)		1.334 (0.490–3.630)		0.919 (0.397–2.128)	

NLR: neutrophil-to-lymphocyte ratio; PLR: platelet-to-lymphocyte ratio; LMR: lymphocyte-to-monocyte ratio: AGR: albumin/globulin ratio

*: *p* value ≤ 0.05

### Prognostic parameters

To evaluate the association of baseline characteristics and prognosis, Kaplan–Meier survival analysis and log-rank tests were performed. The DFS was 78.3%. Clinical parameters for prediction of DFS were further investigated by univariate analysis with Cox regression model. The significantly associated variables were included to perform multivariate Cox regression model. In multivariate analysis treatment history, tumor length, bisphosphonate treatment, NLR and PLR were associated with DFS (*p* < 0.05). In the model of DFS, those factors were verified to be independent prognostic factors in patients with GCT (*p* < 0.05) (Table 4).

**Table 4:** Cox regression model of spinal GCT

	DFS			
	Univariate analysis	*p*	Multivariate analysis	*p*
	HR (95%)		HR (95%)	
**Age (years)**		0.001*	-	-
≤ 60	1.000			
> 60	6.011 (2.618–13.802)			
**Gender**		0.266	-	-
Male	1.000			
Female	0.640 (0.290–1.415)			
Treatment history		0.096		0.013*
Primary	1.000		1.000	
Recurrent	1.963 (0.887–4.343)		3.003 (1.267–7.117)	
**Duration of symptoms**		0.028*	-	-
≤ 12	1.000			
> 12	5.023 (1.192–21.170)			
**Preoperative Frankel score**		0.629	-	-
A-C	1.000			
D-E	0.826 (0.381–1.791)			
**Tumor location**		0.231	-	-
Cervical	1.000			
Thoracic	2.758 (0.759–10.023)			
Lumbar	1.742 (0.462–6.569)			
Sacrum	3.264 (0.843–12.629)			
**Involved segment**		0.520	-	-
Monosegment	1.000			
Multisegment	0.771 (0.349–1.704)			
**Tumor length (cm)**		0.006*		0.024*
≤ 3	1.000		1.000	
> 3	2.820 (1.340–5.931)		2.466 (1.129–5.388)	
**Preoperative embolization**		0.603	-	-
Yes	1.000			
No	1.218 (0.580–2.560)			
**Enneking staging**		0.591	-	-
I	1.000			
II	1.322 (0.407–4.296)			
III	0.771 (0.337–1.761)			
**Bisphosphonate treatment**		0.001*		0.008*
Yes	1.000		1.000	
No	4.466 (1.897–10.513)		3.294 (1.364–7.955)	
**Adjuvant radiotherapy**		0.489	-	-
Yes	1.000			
No	1.314 (0.606–2.848)			
**NLR**		0.001*		0.015*
≤ 2.7	1.000		1.000	
> 2.7	14.895 (3.532–62.816)		6.472 (1.446–28.966)	
**PLR**		0.001*		0.003*
≤ 215.8	1.000		1.000	
> 215.8	7.444 (3.269–16.953)		3.753 (1.576–8.938)	
**LMR**		0.001*	-	-
≤ 2.8	1.000			
> 2.8	0.152 (0.067–0.345)			
**AGR**		0.018*	-	-
< 1.5	1.000			
≥ 1.5	0.356 (1.151–0.839)			

GCT: giant cell tumor; HR: hazard ratio

*: *p* value ≤ 0.05

### Treatment and outcome of recurrent cases

In our series, 28 patients were admitted into our center as recurrent cases. Compared to primary patients, recurrent cases had poorer neurologic status (*P* = 0.048), higher malignant proportion (*P* = 0.002), more blood loss (*P* = 0.016) and transfusion volume (*P* = 0.031), and higher death rate (*P* < 0.0005). The re-recurrence rate of them was 32.1%, while recurrence rate for primary patients was 18.8% (*P* = 0.089). We found that total en bloc spondylectomy could significantly reduce re-recurrence rate in recurrent cases, which was coincident with our former founding [3, 9]. (*P* = 0.025, adjusted *P* = 0.037, OR = 0.0007, Table 5).

**Table 5:** Treatment and outcome of 28 patients with recurrent GCT in the spine

Factors		*n*	Recurrence rate (%)	*p*	Adjusted p	OR
Resection mode	En bloc / Piecemeal	10/15	9.1% vs. 47.1%	0.025	0.037*	0.007
Adjuvant radiotherapy	Yes/No	6/19	14.3% vs. 38.1%	0.334	0.328	
Bisphosphonate treatment	Yes/No	6/19	26.3% vs. 44.4%	0.2	0.352	

OR: Odds ratio

*: *p* value ≤ 0.05

### Nomograms for predicting prognosis of spinal GCT patients

**Figure 2:**
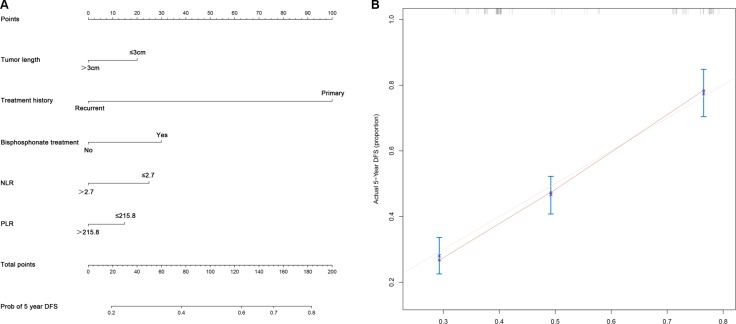
Nomograms convey the results of prognostic models using clinical characteristics and pretreatment inflammatory biomarkers to predict DFS of patients with GCT (**A**) Nomograms can be interpreted by summing up the points assigned to each variable, which is indicated at the top of scale. The total points can be converted to predicted probability of recurrence for a patient in the lowest scale. (**B**) Calibration curves for DFS using nomograms with clinical characteristics and pretreatment inflammatory biomarkers are shown. The Harrell’s c-indexes for OS and DFS prediction were 0.728 (95% CI: 0.710–0.743). The x-axis is nomogram-predicted probability of survival and y-axis is actual survival. The reference line is 45° and indicates perfect calibration.

To predict DFS of patients with GCT, nomogram was established by multivariate Cox regression model according to all significantly independent factors for DFS. Nomogram can be interpreted by summing up the points assigned to each variable, which is indicated at the top of scale. The total points can be converted to predicted probability of recurrence for a patient in the lowest scale. The Harrell’s c-indexes for DFS prediction were 0.728 (95% CI: 0.710–0.743) (Figure 2). Calibration curve for nomogram revealed no deviations from the reference line and no need of recalibration.

## DISCUSSION

Spine is a relatively rare site for GCT but poses great challenge for the treatment of GCT [29]. High recurrence rate is a typical feature of spinal GCT and also an important factor influencing the prognosis. How to prevent postoperative recurrence is a hot issue of spinal GCT. Our former published study has revealed that the surgical method and bisphosphonate are independent prognostic factors [3, 9].

In this research, we reported on a large series of GCTs in the spine treated surgically and aimed to give an answer to those questions:

1) Which parameters have the prognostic value for the predicting GCT recurrence?

2) Dose the inflammatory factors influence the disease progressing?

3) Dose the widely used adjuvant therapies, such as adjuvant radiotherapy, intraoperative local treatment, and bisphosphonate treatment have therapeutic effect?

4) Build the applicable nomogram to evaluate and predict the prognosis of spinal GCT.

Our analysis revealed that treatment history, tumor length, bisphosphonate treatment, NLR and PLR were associated with DFS, which could be the prognostic factor for the spinal GCT. Inflammatory factors such as NLR and PLR does have the influence to the disease prognosis. What’s more, our data indicated that total en bloc spondylectomy was superior to piecemeal total resection in the treatment of recurrent spinal GCT which was coincide with our former published founding [9], while bisphosphonate treatment could serve as a favorable adjuvant therapy for benign GCT in the spine [3, 9].

Treatment history means the therapeutic process patients have experienced before go to our center for surgical treatment. We found that, the retreated group (or the recurrent group) had higher re-recurrent possibility. This phenomenon may associate with the tumor size and surgical procedure. If the tumor broke-through the margin of vertebra or invaded the soft tissue, tumor cell could be residual though gross total resection surgery were made, which may contribute to the poor prognosis. Recurrence of GCT, which may exacerbate the neurologic defects, increase the difficulty of surgery, and even lead to death in cases of lost opportunity to receive surgery again, is a big problem for clinicians [5, 9]. Therefore, the first operation opportunity is precious for both doctors and patients [4].

Inflammation produced by the secretion of cytokines and chemokines promotes tumor growth, angiogenesis and metastasis [12–14, 17]. Several studies have shown that platelets induce circulating tumor cell epithelial-mesenchymal transition and promote extravasation to metastatic sites [15, 22]. Neutrophils promote adhesion and seeding of distant organ sites through the secretion of circulating growth factors such as VEGF and proteases [29, 30]. On the contrary, lymphocytes are basic components of the adaptive and innate immune system and the cellular basis of immune-surveillance and immune-editing, and CD8+ and CD4+ T-lymphocyte interaction among each other could be proven to induce tumor cell apoptosis in antitumor reaction of the immune system [32, 33]. Thus, inflammation induces changes in the cancer microenvironment that favor tumor progression. In light of this, several inflammatory parameters have been investigated as possible predictors of prognosis and response to treatment in different tumor types [22, 24, 34]. Among these, NLR and PLR represent the most common indices [21, 22, 30, 31]. Compared with other potential markers, the measurement of these parameters has the advantage of being inexpensive and reproducible. In this study, NLR and PLR were considered as the independent indicator for DFS of spinal GCT.

Bisphosphonate treatment is used as an adjuvant therapy to control osteolytic lesions of bone tumors in our center [4, 5]. Zoledronic acid and incadronate disodium, which are bisphosphonates, are confirmed to control GCT cells *in vitro* studies [10, 35, 36], and it could significantly relieve cancer pain and the progression of GCT in clinical treatment [32, 33, 37]. Tse et al. reported that bisphosphonate treatment was an effective adjuvant therapy for GCT in the extremity [38]. In this study, we confirmed that it could significantly reduce recurrence rate of benign GCT in the spine, but its positive effect on malignant GCT and recurrent GCT was uncertain. Radiotherapy and chemotherapy are commonly used adjuvant therapy for spinal GCT, but their positive effect on recurrence and overall survival remains controversial [3, 39–41]. Radiotherapy is commonly used to treat cases with intralesional resection and is considered to provide excellent local control of GCT in the extremity [42–44]. However, the risk of post-irradiation sarcoma is a particular concern for patients with spinal GCT [4, 7, 39, 40]. Studies from the Mayo Clinic reported a 17% rate of malignant transformation in previously irradiated GCTs of the spine, sacrum, and pelvis [8]. We also reported 11 patients with secondary malignant GCT in our previous study: 1 of them was confirmed to be radiotherapy-associated and 4 other patients were also received radiotherapy before [4]. In our study, adjuvant radiotherapy could not effectively reduce recurrence rate of spinal GCT. There were also another chemotherapeutics reported to control surgically inaccessible and radio-resistant tumors, but a chemotherapeutic protocol for GCT has not yet been standardized [41, 45].

Recently, breakthroughs have been made in targeted therapy of GCT. Denosumab, a human monoclonal antibody to RANKL (receptor activator of nuclear factor 1 kappa B ligand), has been approved for use in patients with recurrent/unresectable/metastatic GCT or for patients in whom surgery would be morbid [46–48]. But it has some side effects, and several questions remain unclear about the optimal use of this medication [49–51]. As denosumab has not been approved for the treatment of GCT in China, we could not evaluate its effect.

Nomogram have been accepted as reliable tools to integrate important risk factors and predict the outcome for oncology prognosis [20, 52, 53]. And at the same time, the accuracy could be texted by concordance index and calibration curve comparing to other staging systems [28, 33]. More importantly, the graph could provide prognostic information both for groups or individual, which means that it could be used for both doctors and patients to calculate the survival rate [54–56]. In our study, we aim to evaluate the characteristics of GCT patients and try to create a new staging system of nomogram to predict the outcome of the special group. This new method not only reflect the predictive value for each variable but also the complex interaction with the other variables [52]. Moreover, nomogram are the visualizations of the quantized risk variables which was available not only for the surgeons but for each individual patient to understand the short- and long-term outcome.

However, there are still some limitations to this work. The primary weakness of this analysis is its retrospective nature. In addition, there was a selection bias (patients included in the study were all underwent surgery with relatively obvious symptoms; all of recurrence cases in this cohort were taken the first operation in other institutes).

In summary, we confirmed that treatment history, tumor length, bisphosphonate treatment, NLR and PLR were prognostic parameter of spinal GCT. It is the first time to reveal that inflammatory index was associated with the recurrence of GCT. What’s more, the nomogram was established to make easier and more accurate predict for the first time. Bisphosphonate treatment had favorable pain control effect and could serve as an effective adjuvant therapy for benign GCT in the spine. Spinal GCT is a tumor with high recurrence rate, and re-treatment of recurrent cases was companied with more difficulty and high risk. Thus, we should cherish the first operation opportunity, make reasonable evaluation and close fellow-up to realize individualized therapy.

## MATERIALS AND METHODS

### Study population

A retrospective analysis was conducted in patients with spinal GCT in Changzheng Hospital Orthopedic Oncological Center (CHOOC) between January 2005 and October 2015. This research was approved by hospital Ethics Committee, and written informed consent was confirmed from all patients or their legal guardians.

The inclusion criteria were as follows:

1) patients who have made spinal lesions GTR surgery in our center;

2) GCT was confirmed by histopathology;

3) patients had not taken anti-inflammatory medicines or received immunosuppressive therapy including recent steroid exposure, or with chronic inflammatory diseases including autoimmune diseases and infections before operation;

4) patients had not received neoadjuvant therapy;

5) laboratory tests were obtained before surgery.

**Figure 3:**
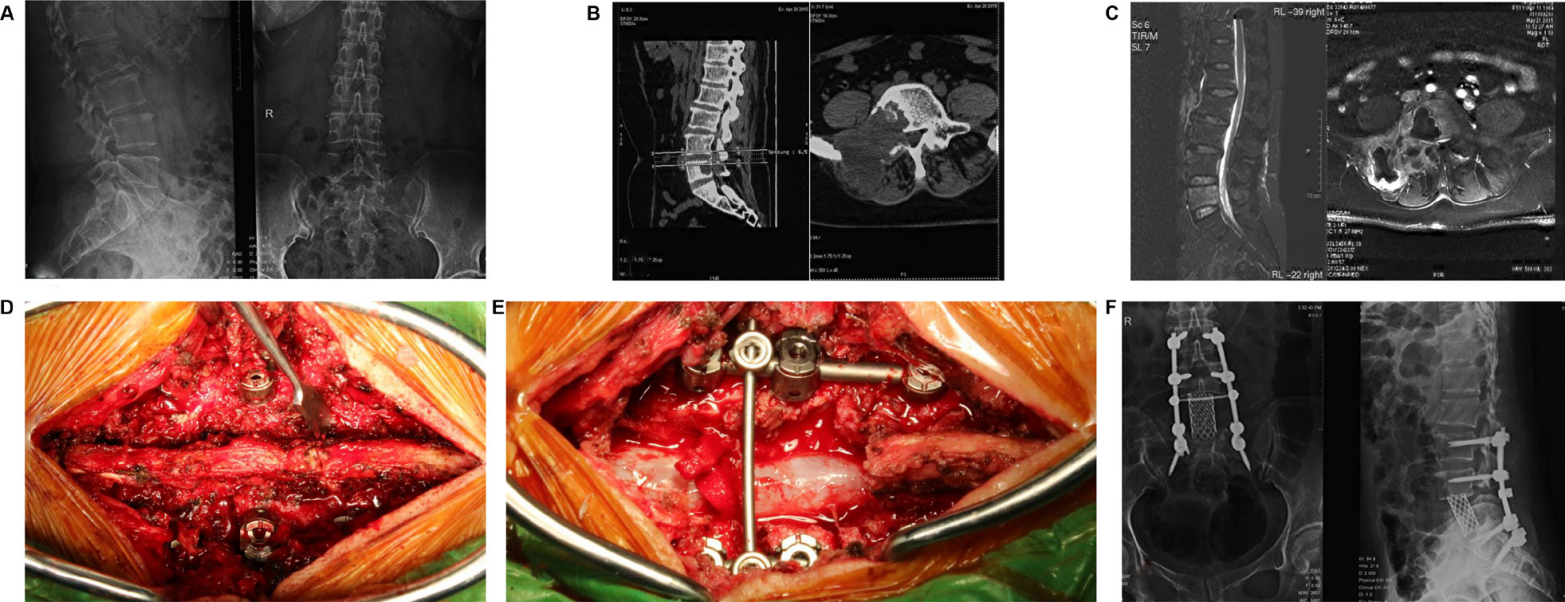
A female patient suffering low back pain for 3 months was made gross total resection (GTR) surgery in Changzheng Hospital Orthopedic Oncological Center (CHOOC) and was pathologically diagnosed as bone Giant Cell Tumor (GCT) (**A**) the pre-surgery X-ray imaging was shown; however the typical “soap bubble changes” was not obvious. (**B**) bone erosion of right part vertebral body was obviously revealed by the computer tomography (CT scan). (**C**) the Magnetic Resonance Imaging (MRI) indicated that the lesion showed low-intensity signal on T1-weighted image and high-intensity signal on T2-weighted image. (**D** and **E**) a gross total resection surgery was conducted; the vertebral body and appendix were removed meanwhile the spine was reconstructed by screw-rod system. (**F**) the post-surgery X-ray imaging showed the L5 vertebra was removed and the internal-fixation was solid and successful.

Finally, a total of 129 patients with spinal GCT accepted total resection at our center between January 2005 and October 2015 were enrolled into study, and the diagnosis of GCT was confirmed by pathology in all patients. The clinical and pathologic data of all patients were retrieved from the maintained medical records in CHOOC. Frankel score was used to evaluate the preoperative neurologic status, and the resected GCTs were classified as benign or malignant according to histological appearance and imaging manifestations. Gross total resection (GTR) was performed in all patients by either piecemeal or en-bloc method. Some patients also received adjuvant therapies, such as adjuvant radiotherapy, intraoperative local treatment, and bisphosphonate treatment. One typical case imaging and therapeutic material was shown in Figure 3.

Death is a rare event for patients with GCT, and we specifically focused on the recurrence status after the initial surgery in our center. All patients were followed up on an outpatient basis at 3, 6, and 12 months after surgery, every 6 months for second year, and then annually for life. The disease free time (DFS) was defined as the interval from the date of surgery to the diagnosis of recurrence. The follow-up period was defined as the interval from surgery to death, or until October 2015 for surviving patients. The recurrence status was confirmed by clinical manifestations and imaging findings in outpatient follow-up, or pathologic evaluation of second surgery. The information of death was acquired through telephone interviews.

### Statistical analysis

Statistical calculations were analyzed using SPSS version 19.0 (SPSS, Inc. Chicago, IL, USA) and R 3.1.2 software (Institute for Statistics and Mathematics, Vienna, Austria). Quantitative data was described by median (range), and qualitative data was described as counts and percentages. X-tile 3.6.1 software20 (Yale University, New Haven, CT, USA) was used to determine the optimal cut-off values for NLR, PLR and LMR. Chi-square test and multivariate logistic regression analysis were used to analyze the relationship between clinical parameters and these inflammatory biomarkers. The DFS were calculated by the Kaplan–Meier method, and the difference of variables was compared using log-rank tests. Univariate analysis was used to examine the association between various prognostic predictors and DFS. Significant prognostic predictors associated with DFS were included to perform multivariate analyses by using the Cox proportional hazards model. *P* values of ≤0.5 were considered statistically significant. All confidence intervals (CIs) were stated at the 95% confidence level. NLR was obtained by dividing the absolute neutrophil count by the absolute lymphocyte count, and PLR was calculated as the ratio of absolute platelet count to absolute lymphocyte count.

Nomograms for possible prognostic factors associated with DFS were established by R software, and the model performance for predicting outcome was evaluated by Harrell’s concordance index (c-index), which is a measure of discrimination. The maximum value of the c-index is 1.0, indicating a perfect discrimination, whereas 0.5 indicates a random chance to correctly discriminate outcome. In addition to measuring discriminative capacity by c-index, each model was evaluated with calibration curve in which predicted outcomes versus observed outcomes are graphically depicted, which made it possible to conduct further comparison of accuracy in estimating prognosis.
